# Announcement: Metals in Biology Gordon Research Conference

**DOI:** 10.1155/MBD.1997.301

**Published:** 1997


					METALS IN BIOLOGY
GORDON RESEARCH CONFERENCE
18-22 Janu.ary 1998,_
Harbortown Hotel, Ventura, California
Conference Chair
Kenneth D. Karlin
Department of Chemistry
Johns Hopkins University
Baltimore, MD 21218-2685, USA
Conference Co-Chair
Eckard Menck
Department of Chemistry
Carnegie-Mellon University
Pittsburgh, PA 15213, USA
PROGRAM:
BIOINORGANIC CHEMISTRY AND BIOGEOCHEMICAL CYCLES
Ed Stiefel (Exxon Company), Bioinorganic chemistry and biogeochemical cycles
IRON SULFUR CLUSTERS
Dimitri Coucouvanis (U. Michigan), Partial structural analogs for t.he nitrogenase co.factor
Barbara Burgess (UC Irvine), The mechanism of Ferredoxin mediated regulation of
NADPH:ferrec3oxin reductase in Azotobacter vinelandii
Lance Seefeldt .(Utah State U.), Electron transfer reactions and MgATP hydrolysis in
nitrogenase catalysis
Joan Broderick (Amherst College), Pyruvate formate lyase activase: Radical generation by
an Fe-S cluster
ZINC PROTEASES
Carl Decicco (Dupont Merck), Zinc metallo.proteinases and drug discovery
Rick Holz (Utah State U.), Mechanistic stud=es on peptide hydrolysis by dinuclear
metalloenzymes
METAL REGULATION AND PROCESSING
Richard Palmiter (HHMI; U. Washington), Vesicular zinc
Tom O'Halloran (Northwestern), Getting metal ions to the right place in the cell: chaperone
proteins and vesicles
Bob Hausinger (Michigan State U.), Nickel incorporation into urease
Jerry Kaplan (U. Utah), Yeast mitochondrial iron metabolism: Insights into human disease
MEDICINE AND DIAGNOSTICS
Steve Lippard (MIT), On the biological mechanism_of cisplatin
Tom Meade (C.al Tech), New MRI contrast agents: Progress towards monitoring gene
exp_ression
1vvo.
PEROXYNIT TE AND NITROGEN OXIDE BIOLOGY/CHEMISTRY
tMhike Stern (Monsanto Company), Peroxynitrite decomposition catalysts. Novel
erapeutics for peroxynitrite-mediated pathology_
John Groves (Princeton U.), Biological targets of peroxynitrite. Redox pathways,
_mechanisms and membrane permeability
Peter Kroneck (U. Konstanz, Germany), Nitrous oxide reductase, a copper enzyme with
novel chromophores
Mike Marietta (HHMI; U. Michigan), Piecing together cofactors and mechanism in nitric
oxide synthase
PHYSICAL STUDIES ON ELECTRON TRANSFER CENTERS
Eckard MLinck (Carnegie-Mellon U.), On the nature of the A- and C-clusters of CO
dehydrogenase
ClaudioLuchinat (U. Florence, Italy), Ferredoxins and cytochromes: beyond the solution
structure
DIOXYGEN PROCESSlNG/O2-ACTIVATION
Dan Stack (Stanford University), Catalytic functional model of galactose oxidase
Ninian Blackburn (Oregon Grad Inst.), Peptidylglycine alpha--hydroxylating monooxygenase:
structure and, mechan=sm.
Chris Schofield (Oxford U., UK), Structures and mechanisms of non-heme iron
oxygenases
Anne-Francis Miller (Johns Hopkins U.), The basis for metal ion specificity in Fe- and Mn-
su_peroxide dismutase
John Caradonna.(Yale U.), Interactions of reduced dioxygen species with non-neme iron
centers: from synthetic models to p_roteins
BIOINORGANIC DISEASE STATES (Joint Session with Graduate Research Seminar (GRS))
Joan Valentine (UCLA), Searching for the link between Cu-Zn superoxide dismutase and ALS
Questions and/or request for Applications:
GRC Web site: http://www.grc.uri.edu GRC E-mail: grc@grcmail.grc.uri.edu
Professor Kenneth D. Karlin
Phone: + 410-516-8027; Fax: + 410-516-6164 (direct]; Fax: + 410-516-8420 (Chem. Dept.]
Email: karlin@jhuvms.hcf.jhu.edu
301

				

